# Supplementary material for: Fast hierarchical processing of orthographic and semantic parafoveal information during natural reading
Source: Nat Commun. 2025 Oct 7;16:8893. doi: 10.1038/s41467-025-63916-y (PMC12504734; doi:10.1038/s41467-025-63916-y)
Supplement: Supplementary file 1 — Supplementary information [file 41467_2025_63916_MOESM1_ESM.pdf]

Supplementary information

Fast hierarchical processing of orthographic and semantic parafoveal information during natural reading

Lijuan Wang[1]*, Steven Frisson[1], Yali Pan[1#] and Ole Jensen[1,2,3#]*

*co-corresponding: ole.jensen@psych.ox.ac.uk; lxw203@student.bham.ac.uk

[#]co-senior authorship

[1]Centre for Human Brain Health, University of Birmingham, Edgbaston, B15 2TT, Birmingham, UK

[2]Department of Experimental Psychology, University of Oxford, Oxford OX2 6GG, UK

[3]Oxford Centre for Human Brain Activity, Wellcome Centre for Integrative Neuroimaging, Department of Psychiatry, University of Oxford, Oxford OX3 7JX, UK

List of Contents

Supplementary Figure 1. Controlled representational similarity analysis for first fixations on pre-target words.

Supplementary Figure 2. The RSA analysis for fixations on target and post-target words.

Supplementary Figure 3. Eye movement characteristics for first fixations on pre-target words.

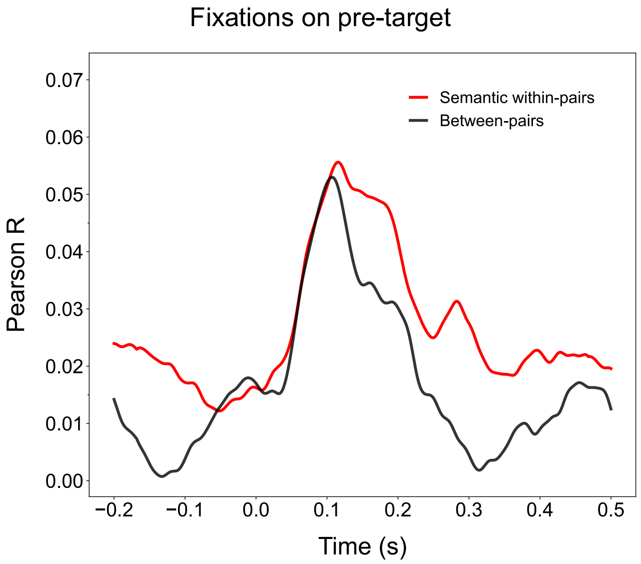

Supplementary Figure 1 | Controlled representational similarity analysis for first fixations on pre-target words.

The time series of representational similarity (Pearson *R*-values) for semantic within-pairs (red line) and between-pairs (black line), with trials excluded where the eyes moved to the target word within 247 ms of pre-target fixation onset. The effect remained significant in the 141–195 ms interval (p = .041, n = 35, two-sided cluster-based permutation test). Source data are provided as a Source Data file.

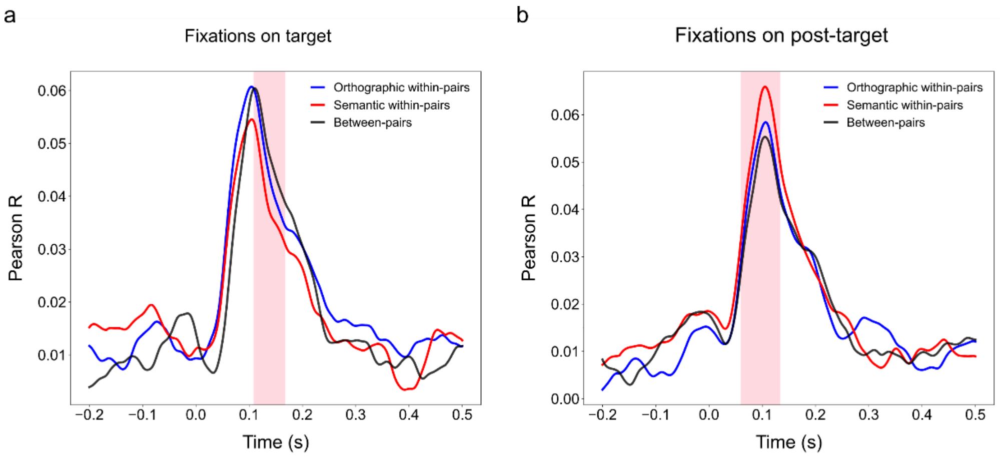

).

Supplementary Figure 2 | The RSA analysis for fixations on target and post-target

(a) Time series of RSA (Pearson's *R* values) aligned to fixation onset on target words. Representational similarity is shown for orthographic within-pairs (blue), semantic within-pairs (red), and between-pairs (black). First, the similarity for orthographic and

semantic within-pairs exceeded the between-pairs at about 60–80 ms after fixation onset on the target word, while these effects did not reach statistical significance (orthographic: 60–89 ms, $p = 0.083$; semantic: 60–76 ms, $p = 0.189$; $n = 35$, two-sided cluster-based permutation test). Later, in the 109 – 167 ms interval (indicated by light red shading), the semantic within-pairs produced significantly higher R values than the between pairs ($p = .023$, $n = 35$, two-sided cluster-based permutation test). (b) Time series of RSA aligned to fixation onset on post-target words. No significant difference was observed between orthographic within-pairs and between-pairs (74–80 ms, $p = 0.29$; $n = 35$, two-sided cluster-based permutation test). However, semantic within-pair similarity was significantly higher than between-pair similarity during the 60–133 ms interval (shaded in light red; $p = 0.008$, $n = 35$, two-sided cluster-based permutation test).Source data are provided as a Source Data file.

The dynamics of word representations

To examine whether the observed effects persisted during fixations on the target word, we conducted supplementary RSA analyses time-locked to the fixation onset on the target word. First, we observed that the similarity for orthographic and semantic within-pairs exceeded the between-pairs at about 60–80 ms after fixation onset on the target word, while these effects did not reach statistical significance (orthographic: 60–89 ms, $p = 0.083$; semantic: 60–76 ms, $p = 0.189$; $n = 35$, two-sided cluster-based permutation test). Then, the orthographic effects were no longer observed as the orthographic within and between-pair similarities became comparable. This suggests that orthographic information extraction has already been completed during parafoveal processing and decayed thereafter, as it becomes irrelevant for ongoing sentence comprehension. Interestingly, we observed an unexpected reversal effect at about 100 ms: semantically related target words (e.g., "writer" vs "author") exhibited lower neural representational similarity than unrelated pairs, with a significant difference in the 109–167 ms interval ($p = .021$, $n = 35$, two-sided cluster-based permutation test). A possible explanation could be related to the depth of processing. Parafoveal semantic processing is coarse and likely at gist level. For example, the parafoveal words "writer" and "author" might

be represented as “someone who produces written text” with similar neuronal representations. However, when these words are fixated, more detailed semantic processing is allowed, enabling finer differentiation between similar concepts. For example, a “writer” might refer to a person who has typed some text; while an “author” might refer to someone who has published written material. Consequently, during foveal processing, the neural “representational distance” between "writer" and "author" should be greater, even more so than that of dissimilar words, to ensure they are sufficiently differentiated from each other. Notably, this reversed pattern was specific to the target fixation: when analyses were time-locked to the subsequent fixation (i.e., after the target word), semantic similarity effects reappeared in the expected direction, with higher similarity for within-pairs than between-pairs (60–133 ms; $p = .011$, $n = 35$, two-sided cluster-based permutation test). This dynamic change in word representations indicates that words are processed at different levels across several saccades, which may optimise the information extraction from words. However, the above explanation is tentative and needs further exploration.

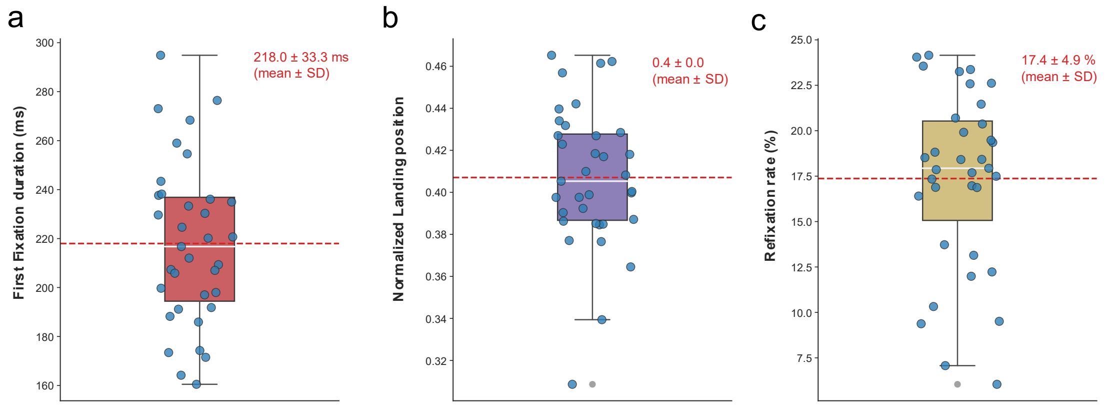

Supplementary Figure 3 | Eye movement characteristics for fixations on pre-target words.

(a) The first fixation durations on the pre-target words. Each dot represents the mean first fixation duration for an individual participant. The red dashed line indicates the grand average (n = 35). The box represents the interquartile range (IQR), with the central line marking the median. Outliers are shown as grey circles. (b) The average landing position on first fixations on pre-target words (normalised from 0 to 1 according

to physical word length). (c) Refixation rate (%) of pre-target words. Source data are provided as a Source Data file.
