# Supplementary material for: Fast hierarchical processing of orthographic and semantic parafoveal information during natural reading
Source: Nat Commun. 2025 Oct 7;16:8893. doi: 10.1038/s41467-025-63916-y (PMC12504734; doi:10.1038/s41467-025-63916-y)
Supplement: Supplementary file 2 — Reporting Summary [file 41467_2025_63916_MOESM2_ESM.pdf]

nature portfolio

Corresponding author(s): Prof Ole Jensen; Miss Lijuan Wang

Last updated by author(s): 25/07/25

Reporting Summary

Nature Portfolio wishes to improve the reproducibility of the work that we publish. This form provides structure for consistency and transparency in reporting. For further information on Nature Portfolio policies, see our Editorial Policies and the Editorial Policy Checklist.

Statistics

For all statistical analyses, confirm that the following items are present in the figure legend, table legend, main text, or Methods section.

n/a	Confirmed	
☐	☒	The exact sample size (*n*) for each experimental group/condition, given as a discrete number and unit of measurement
☐	☒	A statement on whether measurements were taken from distinct samples or whether the same sample was measured repeatedly
☐	☒	The statistical test(s) used AND whether they are one- or two-sided<br>*Only common tests should be described solely by name; describe more complex techniques in the Methods section.*
☒	☐	A description of all covariates tested
☐	☒	A description of any assumptions or corrections, such as tests of normality and adjustment for multiple comparisons
☐	☒	A full description of the statistical parameters including central tendency (e.g. means) or other basic estimates (e.g. regression coefficient) AND variation (e.g. standard deviation) or associated estimates of uncertainty (e.g. confidence intervals)
☐	☒	For null hypothesis testing, the test statistic (e.g. *F*, *t*, *r*) with confidence intervals, effect sizes, degrees of freedom and *P* value noted<br>*Give P values as exact values whenever suitable.*
☒	☐	For Bayesian analysis, information on the choice of priors and Markov chain Monte Carlo settings
☒	☐	For hierarchical and complex designs, identification of the appropriate level for tests and full reporting of outcomes
☐	☒	Estimates of effect sizes (e.g. Cohen's *d*, Pearson's *r*), indicating how they were calculated

Our web collection on statistics for biologists contains articles on many of the points above.

Software and code

Policy information about availability of computer code

Data collection	Stimuli presentation was coded using MATLAB-based (R2019b; MathWorks Inc., USA) Psychophysics Toolbox -3 (http://psychtoolbox.org/).<br>MEG data were acquired using a 306-sensor TRIUX Elekta Neuromag system (Elekta, Finland).<br>Eye movement data were acquired from eyeLink 1000 Plus (SR Research Ltd, Canada)<br>Structural MRI images were acquired using a 3T Siemens Prisma scanner (Siemens, Germany).
Data analysis	The data analyses were performed in Python (3.10.8) by using mne (version 1.3.0 https://mne.tools/stable/index.html) and custom-made scripts.<br><br>The code used for analyses is available at OSF(https://osf.io/rnx89/)

For manuscripts utilizing custom algorithms or software that are central to the research but not yet described in published literature, software must be made available to editors and reviewers. We strongly encourage code deposition in a community repository (e.g. GitHub). See the Nature Portfolio guidelines for submitting code & software for further information.

Data

Policy information about availability of data

All manuscripts must include a data availability statement. This statement should provide the following information, where applicable:

- Accession codes, unique identifiers, or web links for publicly available datasets
- A description of any restrictions on data availability
- For clinical datasets or third party data, please ensure that the statement adheres to our policy

We will deposit the following data in the current study on figshare repository (https://figshare.com/account/home#/projects/117885), DOI: https://doi.org/10.6084/m9.figshare.27189843
the raw MEG data, the epoch data after pre-processing, the raw EyeLink files, the Psychotoolbox data, and the head models after the co-registration of anatomical MRI with the MEG data.

Research involving human participants, their data, or biological material

Policy information about studies with human participants or human data. See also policy information about sex, gender (identity/presentation), and sexual orientation and race, ethnicity and racism.

Reporting on sex and gender	24 out of 39 participants identified themselves as female sex. No sex or gender-based analyses were performed as these factors were not the focus of the study.
Reporting on race, ethnicity, or other socially relevant groupings	We recruited native English speakers for this natural reading study. As a result, the majority of participants were White; however, race and ethnicity were not criteria for selection. The study also included individuals from other ethnic backgrounds who have spoken English as their primary language since early childhood.
Population characteristics	We recruited thirty-nine native English speakers (28 females), aged 18-35 years old (21 ± 2.3, mean ± SD), right-handed, with normal or corrected-to-normal vision, and without a neurological history or language disorder diagnosis.
Recruitment	We recruited participants through an email list used for recruiting participants for the centre for Human Brain Health (CHBH). All participants who meet the requirements mentioned above would be included without any self-selection bias or other biases. Participants provided written informed consent and were compensated £15 per hour for their participation.
Ethics oversight	The University of Birmingham Ethics Committee approved the study.

Note that full information on the approval of the study protocol must also be provided in the manuscript.

Field-specific reporting

Please select the one below that is the best fit for your research. If you are not sure, read the appropriate sections before making your selection.

[x] Life sciences [ ] Behavioural & social sciences [ ] Ecological, evolutionary & environmental sciences

For a reference copy of the document with all sections, see nature.com/documents/nr-reporting-summary-flat.pdf

Life sciences study design

All studies must disclose on these points even when the disclosure is negative.

Sample size	Previous studies utilizing Representational Similarity Analysis typically recruited 32-34 participants (See below). In our research, the natural reading paradigm led to word skipping, which reduced the number of usable trials compared to these earlier studies. So in the current study, we aimed for at least 35 subjects that can be used for statistical test.<br><br>Wang, L., Jensen, O., & Kuperberg, G. R. (2018). Neural evidence for prediction of animacy features by verbs during language comprehension: Evidence from MEG and EEG Representational Similarity Analysis. In 10th Annual Meeting of the Society for the Neurobiology of Language, Quebec City, Canada.<br>Wang, L., Kuperberg, G., & Jensen, O. (2018). Specific lexico-semantic predictions are associated with unique spatial and temporal patterns of neural activity. elife, 7, e39061.<br>Hubbard, R. J., & Federmeier, K. D. (2021). Representational pattern similarity of electrical brain activity reveals rapid and specific prediction during language comprehension. Cerebral Cortex, 31(9), 4300-4313.
Data exclusions	Four out of thirty-nice subjects were excluded from analysis due to poor eye tracking accuracy, large body movement during experiment, and too many noisy sensors, which left thirty-nine participants.
Replication	The results presented in this article are based on a single experiment. The experiment was not replicated due to substantial time required for data collection and analysis. The study design included a sufficiently powered sample(n=35) and is intended as a foundation for future replication efforts.

Randomization	In the current study, each participant read all sentences, which was a within subject design.<br>We only allocated participants for one of the two versions of sentences, and this was done randomly.
Blinding	There are no distinct groups in this study. The data from all subjects is analyzed using the same software, algorithms and settings.

Reporting for specific materials, systems and methods

We require information from authors about some types of materials, experimental systems and methods used in many studies. Here, indicate whether each material, system or method listed is relevant to your study. If you are not sure if a list item applies to your research, read the appropriate section before selecting a response.

Materials & experimental systems

n/a	Involved in the study
☒	☐ Antibodies
☒	☐ Eukaryotic cell lines
☒	☐ Palaeontology and archaeology
☒	☐ Animals and other organisms
☒	☐ Clinical data
☒	☐ Dual use research of concern
☒	☐ Plants

Methods

n/a	Involved in the study
☒	☐ ChIP-seq
☒	☐ Flow cytometry
☐	☒ MRI-based neuroimaging

Plants

Seed stocks	*Report on the source of all seed stocks or other plant material used. If applicable, state the seed stock centre and catalogue number. If plant specimens were collected from the field, describe the collection location, date and sampling procedures.*
Novel plant genotypes	*Describe the methods by which all novel plant genotypes were produced. This includes those generated by transgenic approaches, gene editing, chemical/radiation-based mutagenesis and hybridization. For transgenic lines, describe the transformation method, the number of independent lines analyzed and the generation upon which experiments were performed. For gene-edited lines, describe the editor used, the endogenous sequence targeted for editing, the targeting guide RNA sequence (if applicable) and how the editor was applied.*
Authentication	*Describe any authentication procedures for each seed stock used or novel genotype generated. Describe any experiments used to assess the effect of a mutation and, where applicable, how potential secondary effects (e.g. second site T-DNA insertions, mosiacism, off-target gene editing) were examined.*

Magnetic resonance imaging

Experimental design

Design type	Resting state
Design specifications	N/A
Behavioral performance measures	No behavioral performance involved.

Acquisition

Imaging type(s)	Structural MRI (T1 scans)
Field strength	3T
Sequence & imaging parameters	Time: 4:52<br>Voxel dimensions: 1mm isotropic<br>TR: 2000ms<br>TE: 2.01ms<br>TI: 880ms<br>Flip angle: 8deg<br>FOV: 256x256x208mm<br>GRAPPA factor: 2
Area of acquisition	A whole brain scan was used.

Diffusion MRI ☐ Used ☒ Not used

Preprocessing

Preprocessing software	FreeSurfer (version 6)
Normalization	To perform group-level analysis, individual source estimates were morphed onto a common source space (i.e., “fsaverage”) .
Normalization template	'fsaverage' in Freesurfer
Noise and artifact removal	N/A
Volume censoring	N/A

Statistical modeling & inference

Model type and settings	N/A
Effect(s) tested	N/A

Specify type of analysis: [x] Whole brain [ ] ROI-based [ ] Both

Statistic type for inference (See Eklund et al. 2016)	N/A
Correction	N/A

Models & analysis

n/a	Involved in the study
[x]	[ ] Functional and/or effective connectivity
[x]	[ ] Graph analysis
[x]	[ ] Multivariate modeling or predictive analysis
